# From Sound Perception to Automatic Detection of Schizophrenia: An EEG-Based Deep Learning Approach

**DOI:** 10.3389/fpsyt.2021.813460

**Published:** 2022-02-17

**Authors:** Carla Barros, Brian Roach, Judith M. Ford, Ana P. Pinheiro, Carlos A. Silva

**Affiliations:** ^1^Psychological Neurosciences Lab, Psychology Research Center (CIPsi), School of Psychology, University of Minho, Braga, Portugal; ^2^Psychiatry Service, San Francisco Veteran Affairs Medical Center (VAMC), San Francisco, CA, United States; ^3^Department of Psychiatry, University of California, San Francisco, San Francisco, CA, United States; ^4^Research Center for Psychological Science (CICPSI), Faculdade de Psicologia, Universidade de Lisboa, Lisbon, Portugal; ^5^Center for MicroElectromechanical Systems (CMEMS-UMinho), University of Minho, Guimarães, Portugal; ^6^LABBELS - Associate Laboratory, Guimarães, Portugal

**Keywords:** auditory processing, convolutional neural network, deep learning, EEG, schizophrenia

## Abstract

Deep learning techniques have been applied to electroencephalogram (EEG) signals, with promising applications in the field of psychiatry. Schizophrenia is one of the most disabling neuropsychiatric disorders, often characterized by the presence of auditory hallucinations. Auditory processing impairments have been studied using EEG-derived event-related potentials and have been associated with clinical symptoms and cognitive dysfunction in schizophrenia. Due to consistent changes in the amplitude of ERP components, such as the auditory N100, some have been proposed as biomarkers of schizophrenia. In this paper, we examine altered patterns in electrical brain activity during auditory processing and their potential to discriminate schizophrenia and healthy subjects. Using deep convolutional neural networks, we propose an architecture to perform the classification based on multi-channels auditory-related EEG single-trials, recorded during a passive listening task. We analyzed the effect of the number of electrodes used, as well as the laterality and distribution of the electrical activity over the scalp. Results show that the proposed model is able to classify schizophrenia and healthy subjects with an average accuracy of 78% using only 5 midline channels (Fz, FCz, Cz, CPz, and Pz). The present study shows the potential of deep learning methods in the study of impaired auditory processing in schizophrenia with implications for diagnosis. The proposed design can provide a base model for future developments in schizophrenia research.

## 1. Introduction

Schizophrenia (SZ) is a chronic and complex brain disorder that affects social and cognitive functioning (1). SZ is characterized by the presence of positive (e.g., hallucinations and delusions) and negative symptoms (e.g., blunted affect), as well as cognitive deficits (2). About 75% of patients experience hallucinations in the auditory modality, most frequently as voices (3). Deficits in auditory processing have frequently been reported in SZ, which may reflect auditory cortex pathology (4). Some studies have documented larger deficits in patients with (vs. without) auditory hallucinations (5).

Several studies aimed to probe the neural mechanisms underpinning auditory processing abnormalities in SZ, using different techniques such as electroencephalography (EEG). Event-related potentials (ERP) of the EEG represent the averaged electrical activity elicited in response to an event (e.g., stimulus, motor response). They provide a suitable method to specify the time course of brain activity in response to auditory stimulation, for example (6). Alterations in ERP components have been consistently documented in SZ and proposed as potential biomarkers of this disorder (7). For example, the mismatch negativity (MMN) and P300 amplitudes are robustly attenuated in SZ (8, 9). In turn, sensory gating studies have shown reduced P50 suppression in SZ patients in auditory paired-stimulus paradigms (10–13). Other studies have focused on the N100 ERP component, specifically showing generalized amplitude reductions in response to sounds in SZ. This reduction seems to be more pronounced in patients who experience auditory hallucinations (14, 15).

Deep learning applied to EEG data could represent a promising contribution to a more accurate prediction of psychosis conversion in at-risk states of or treatment response in diagnosed patients, as well as of disease trajectories (16). EEG-based deep learning algorithms have also seen growing interest among neuroscientists, especially in the context of brain-signal decoding. However, deep learning is still a poorly explored method in the development of EEG-based models applied to SZ diagnosis and prediction. Convolutional networks have been implemented with the goal of recognizing and classifying patterns from multivariate time series, such as the EEG signal (17–19). The success of this type of neural network applied to EEG data for decoding purposes has prompted us to investigate the use of convolutional neural networks (CNN) for SZ classification.

### 1.1. Related Work

SZ classification on the basis of early auditory EEG-derived ERP components has already been attempted with classical machine learning models (20–22). Components such as the P300, MMN, or N100 were mainly elicited with auditory oddball and passive listening paradigms and used as input features in SZ recognition with random forest (RF), support vector machine (SVM), or linear discriminant analysis (LDA) classifiers. The results of these studies underscore the potential of auditory ERP components recurrently proposed as SZ biomarkers when their characteristics are used as features to discriminate patients from healthy subjects. A limitation of these approaches relates to the process underlying feature extraction and selection, which requires domain expertise. The few studies that probed the potential of deep learning in EEG-based classification of SZ (23–27) achieved the best performances with CNN-based models applied to resting-state EEG data, which is independent of cognitive or sensory processing. Despite their capacity to discriminate healthy from SZ subjects, these models do not inform about auditory processing, which is affected in SZ (4). Very recently, Aristizabal et al. (28) explored both machine and deep learning techniques to identify children at risk of SZ on the basis of EEG data collected during a passive auditory oddball task. In the classical machine learning approach, the mean amplitude was extracted in the 80–220 ms and 160–290 ms latency intervals, when ERP components indexing of sensory processing (N100 and P200, respectively) are expected to emerge. The mean values were extracted for 5 midline electrodes: Fz, FCz, Cz, CPz, and Pz. Using common classifiers, such as decision trees, k-nearest neighbors, and SVM, the discrimination between healthy and at-risk children based on those features was unsuccessful. As for the deep learning approach, a 2D-CNN-LSTM was proposed, composed of one 2D convolutional layer, followed by normalization and fully-connected layers. The information from the previous block was processed with a stack of two LSTM (long-short term memory) networks, whose output was transformed with sigmoid non-linearity for classification purposes. This model based on EEG single-trials achieved the best performance in at-risk children identification. For each trial, a spatio-temporal 2D signal was created using a 300 ms post-stimulus window focusing on the 5 midline electrodes. The machine learning attempt illustrates the difficulty in specifying stimulus-related signal features that allow a precise identification of SZ risk. The results of both approaches may reflect the developmental phase of the population under study, namely ongoing developmental brain maturation processes. Notwithstanding, this study demonstrates the potential of deep learning methods in subjects' discrimination as a function of psychosis risk.

Evidence for altered auditory processing in SZ has fostered the investigation of the dynamics of electrical brain activity targeting the differentiation between patients and healthy subjects. Amplitude reduction of auditory evoked potentials such as the N100 have been consistently reported in the literature (14, 29). Those alterations have driven the use of machine learning methods for automatic SZ recognition. Beyond the time-consuming feature extraction, both machine learning models and ERP analysis exhibit a major limitation: the non-uniformity of the time windows and electrodes used for feature selection across studies. By contrast, deep learning methods profit from automatic pattern learning, with minimal human intervention. Although deep learning architectures based on EEG signals have been proposed for SZ classification, the learning of patterns from the electrical brain response to auditory stimuli is a scarcely investigated topic. A recent review provided a critical analysis of deep learning and classical machine learning methods to detect SZ based on EEG signals (30), highlighting the potentialities of these methods in clinical research. Notwithstanding, from this review it is also clear that more studies are necessary and that surpass the limitations of the existing ones. The current work intends to assess the potential of deep models to learn discriminatory EEG patterns in the early stages of auditory processing, which may inform about the significance of sensory changes to SZ diagnosis and prognosis. We followed good practices for the development and implementation of machine learning methods proposed in Barros et al. (30).

### 1.2. Contributions

This paper presents a multi-channel deep convolutional neural network for SZ and healthy control (HC) single-trial EEG classification across subjects. The CNN-based architecture is proposed for trial-wise decoding of EEG signals elicited in response to externally generated auditory stimulation. The main contributions of this work are:

- The application of deep learning methods to SZ classification using stimulus-related EEG single-trials recorded during auditory processing of pure tones. Unlike previous deep learning research using resting-state EEG data for the same purpose, our model is based on sensory processing measures. Since auditory impairments have been documented in SZ, we hypothesize that the signal's dynamic during sound processing is altered in this disorder.
- The recording of EEG data during a passive listening task to provide discriminatory information. The proposed task is of short duration, easily implemented in a clinical environment, and does not require an overt response from the subject.
- The specification of which number of electrodes and which scalp localization should be used to most accurately predict whether the signal belongs to a SZ subject or not. This study sheds light on which electrode location is most relevant for identifying changes in brain activity during auditory processing since most studies in this field use a different number and combination of electrodes.
- The comparison of classical machine learning with deep learning architectures to classify SZ based on auditory EEG responses. Given the advantages of deep learning over machine learning methods, we hypothesize that deep neural networks will be able to learn different patterns based on auditory EEG signals, allowing a more accurate classification of SZ subjects.
- The use of ensemble methods to reduce the variance of predictions of the developed deep learning model.

This paper is organized as follows. The methodology used for data acquisition and the deep learning model's architecture are detailed in Section 2. Section 3 describes the dataset, the evaluation procedure, and the experimental setup. In Section 4, the results are described and discussed. Finally, Section 5 presents the main conclusions.

## 2. Methodology

In this section, we describe the methodology used for EEG data acquisition. With a simple listening task, we aimed to elicit auditory evoked potentials that shed light on the time course of auditory processing. The signal dynamics elicited by auditory stimuli is also explained, showing the expected pattern in both control and SZ subjects. Afterward, the proposed deep learning architecture is presented in detail, which aims to extract information from the time course of the EEG signal and its topographical distribution. The main objective is to obtain a model capable of recognizing patterns in the electrical brain activity underlying auditory processing in SZ. Lastly, the traditional machine learning approaches commonly found in other proposals are introduced. The implementation of these algorithms provides a strong base model for a proper analysis of the specific advantages of deep learning (over machine learning) methods in predicting whether an individual suffers from SZ or not, on the basis of auditory processing alterations.

### 2.1. EEG Data Acquisition

**Figure 1:**
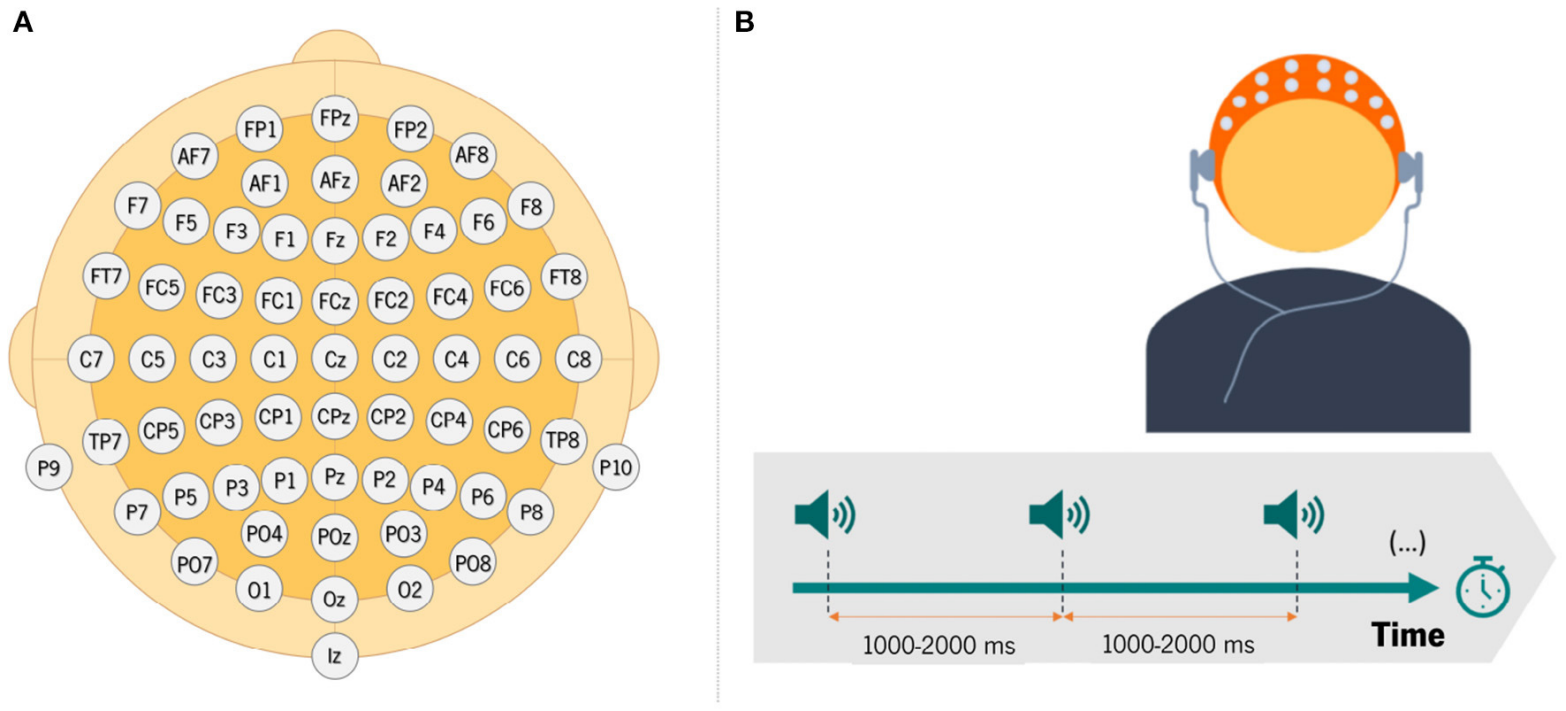
**(A)** Illustration of electrodes placement over the scalp for EEG data recording; **(B)** schematic illustration of the auditory-only condition of the experimental paradigm.

EEG data were recorded using 64 electrodes according to the 10-10 international system configuration, while subjects performed an auditory task (see Figure 1A). After EEG signal acquisition, offline pre-processing was performed: the signal was filtered, segmented, and carefully inspected to remove potential artifacts.

#### 2.1.1. EEG Task

The task involved the presentation of 100 tones, with a variable inter-stimulus interval (ISI—temporal interval from the offset of one stimulus to the onset of another) ranging between 1,000 and 2,000 ms (Figure 1B). The presentation of each sound is called a trial. During data acquisition, the EEG signals were obtained over electrodes placed on the scalp of the participant, who were asked to listen passively to the sounds. This task allows examining the response of the auditory system to externally generated sounds.

#### 2.1.2. Auditory ERP Components

The N100 and the P200 components are typically elicited in response to sound onset. The N100 can be elicited by any discernible auditory stimulus irrespective of task demands (14). This negative potential typically occurs between 80 and 120 ms after sound onset, with maximal amplitude over fronto-central and central electrodes (6, 31). The P200 is a positive deflection that occurs approximately 200 ms after sound onset (6). This component is distributed over centro-parietal electrodes. Its amplitude is generally maximal over the vertex (6, 32). Both N100 and P200 auditory components measured during passive listening tasks can reflect early automatic attention allocation and stimulus categorization, respectively (33). Considering the consistently reported alterations in the N100 and P200 amplitudes in psychosis (14, 32), an approach focused on the time window of these early auditory processing indices has the potential to accurately discriminate HC and SZ subjects.

**Figure 2:**
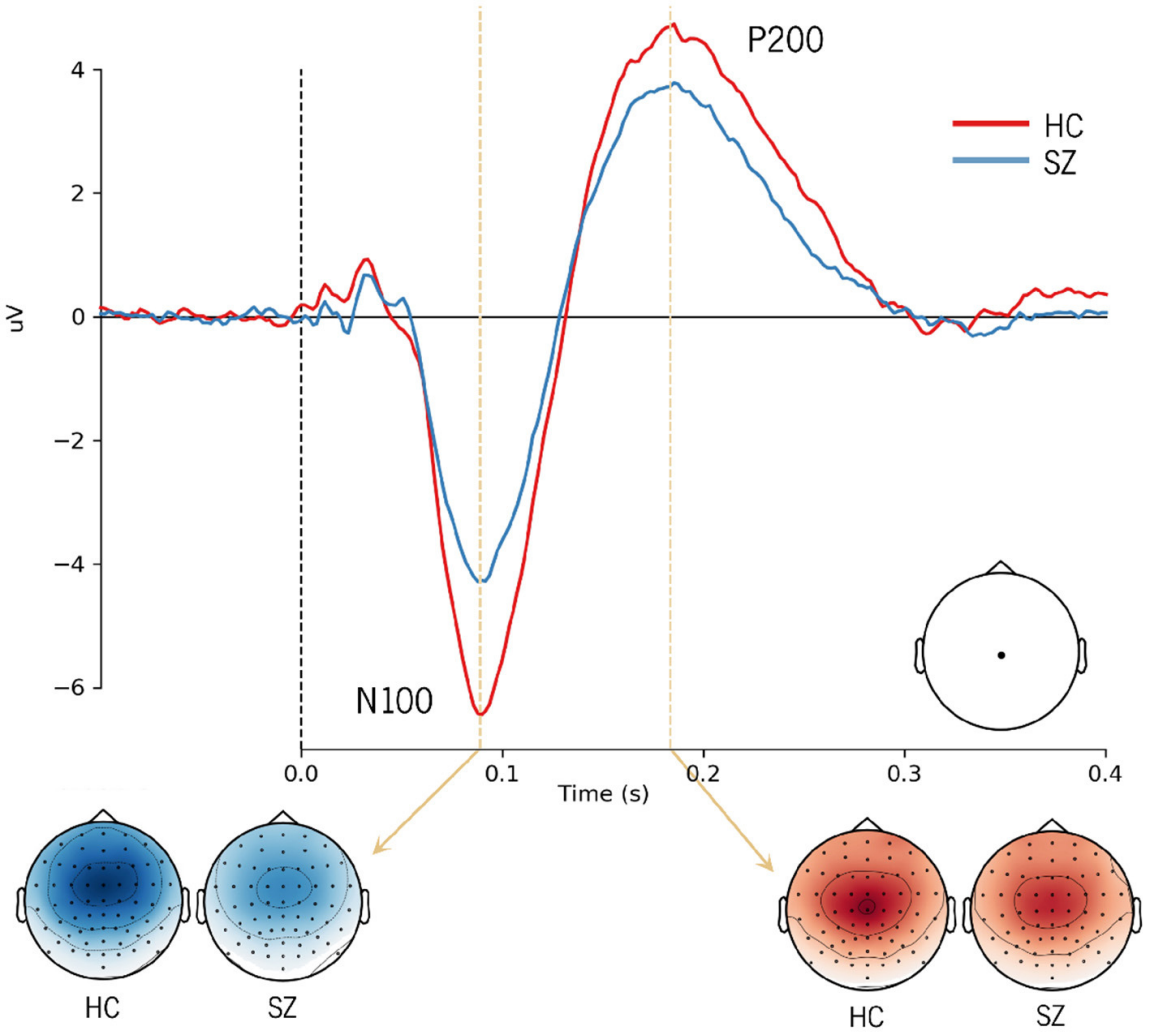
Grand average ERP waveforms showing the N100 and P200 responses to external sounds over Cz electrode in healthy subjects and SZ patients. The topographic distribution of the N100 and P200 components is also shown in both groups.

The ERP analysis consists of averaging all segments related to a given condition from a subject and subsequently computing the grand averages, which represent the average of EEG activity across subjects. When conducting this traditional analysis, the background activity (unrelated to the stimulus) is faded away and the N100 and P200 components emerge. The typical grand averages waveforms obtained from the ERP analysis are illustrated in Figure 2, showing a reduction in the amplitude of the N100 and P200 in SZ. Although the main assumption of signal averaging is that the EEG signal in each trial has stable characteristics, such as morphology, the truth is that ERP averaging hides the intertrial variability in latency and amplitude of the underlying components (34). Therefore, the averaged ERP signals may only represent an approximate picture of the neural processes elicited by an auditory stimulus. Thus, we adopted a single-trial approach, considering the above-mentioned trial-to-trial variability.

### 2.2. Deep Learning Architecture

In the current work, we propose a CNN-based deep learning architecture for SZ classification. The model takes the EEG single-trial recordings as input and provides the probability that a given segment comes from a SZ or HC subject. The acronym SzNet will be used hereinafter to refer to the proposed model.

CNNs are able to find patterns by convolving a filter, or kernel, over the data. Depending on the data structure, the convolutional process can occur in different dimensions. A 1D convolution layer creates a kernel that slides over a single dimension. This can be relevant to find correlations between points in the temporal course or topographical distribution of the EEG data. On the other hand, the use of 2D convolutions allows correlating temporal and spatial information, ensuring that patterns are learned from both dimensions. The maximum signal amplitudes can change in their topographical distribution over time. This is illustrated in Figure 2 by differences in the scalp distribution of the auditory N100 (blue) and P200 (red) peaks reported in the literature, with dark colors corresponding to their maximal activity (N100 - fronto-central distribution; P200 - central distribution).

**Figure 3:**
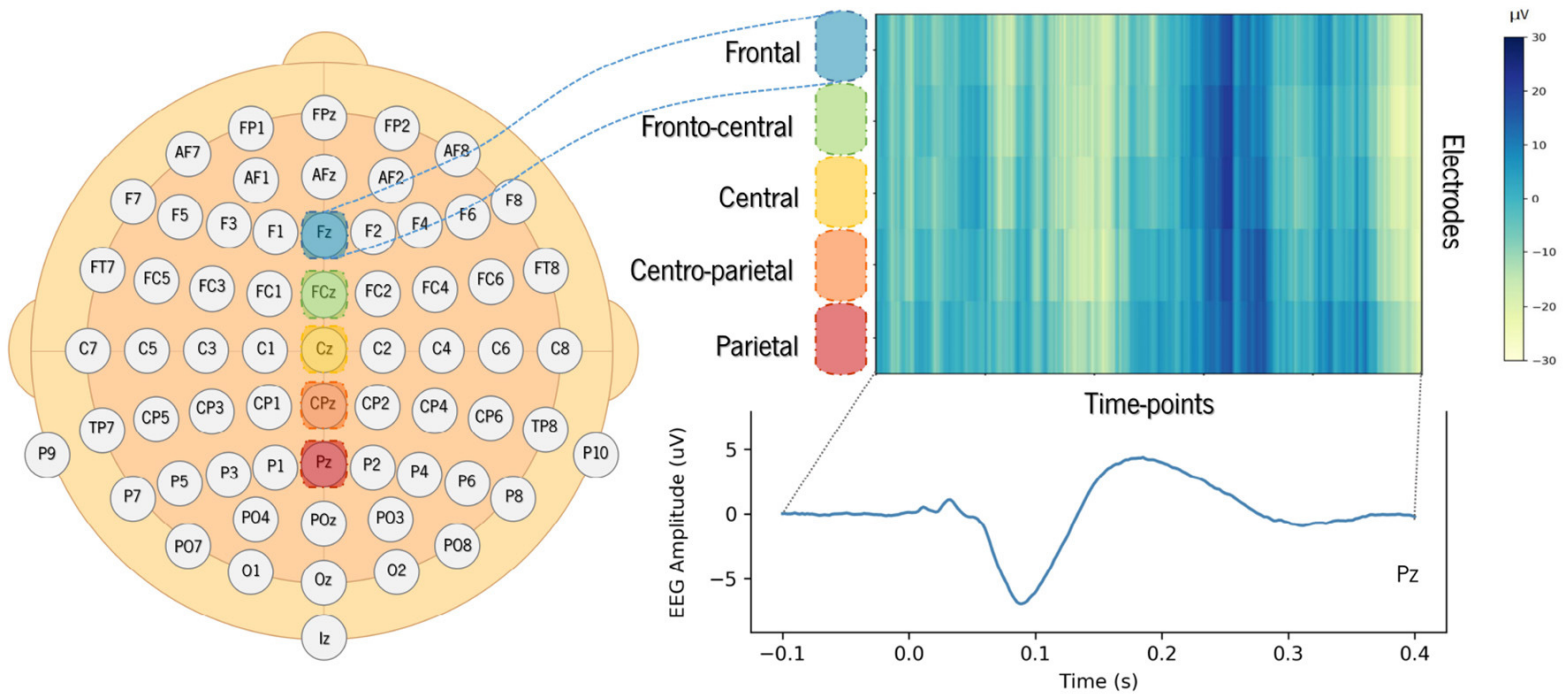
Example of an EEG compounded 2D signal created from one single trial using a set of 5 electrodes. The color scale shows amplitude, in microvolts, over the time points recorded for each of the selected electrodes.

Therefore, a 2D convolutions strategy allows extracting information about amplitude variation over time and, simultaneously, across electrodes. In order to take advantage of bidimensional convolutions, we created a 2D structure for each trial by stacking 1D signals captured from midline electrodes over frontal (Fz), fronto-central (FCz), central (Cz), centro-parietal (CPz), and parietal (Pz) regions of the scalp. We obtained spatio-temporal 2D signals for the EEG segments in which the rows correspond to the selected electrodes, and the columns to the time points of the segment window considered, as illustrated in Figure 3. The value of each coordinate corresponds to the amplitude of the signal. The electrodes (rows) were stacked from the frontal to the parietal regions in the images created, so that the network could extract features with functional meaning.

**Figure 4:**
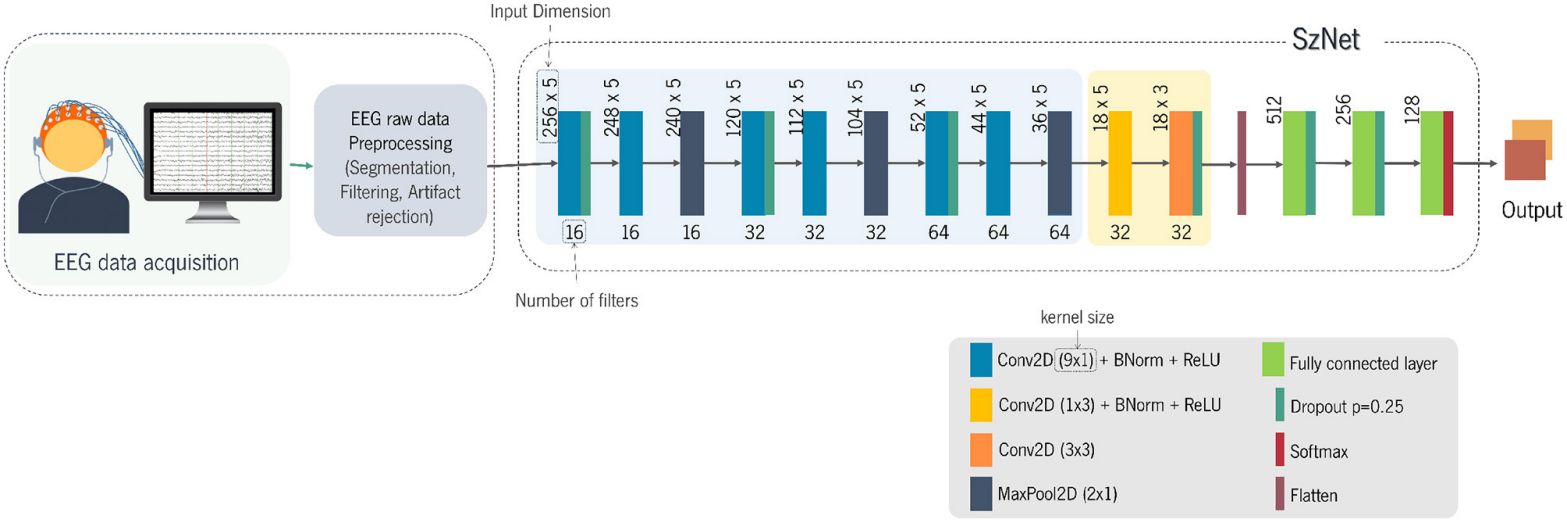
Schematic illustration of the end-to-end process with the first block corresponding to EEG data acquisition and pre-processing. The second block shows the architecture of the SzNet model in detail.

A schematic representation of the main blocks of the proposed method is presented in Figure 4. The first block corresponds to EEG data acquisition and preprocessing, and the second one corresponds to the detailed architecture of the SzNet model. The SzNet architecture is composed of three main types of layers: convolutional, pooling, and fully-connected (FC) layers. Those layers are stacked to increase the network depth, enhancing the selectivity and the sensitivity to slightly relevant variations (35). The learning process of the network is divided into two phases. The first (blue-shaded area in Figure 4) involves learning patterns from the time course of each EEG segment for each electrode. The second (orange-shaded area in Figure 4) aims to correlate those patterns between different electrodes of interest, extracting spatial information. This approach intended to mimic the traditional analysis of this type of data.

The proposed model consists of a stack of 14 layers of neural network connections. Layers 1, 2, 4, 5, 7, and 8 perform 9-point 1D convolutions (9x1 shaped kernels) over the time dimension. This small-size kernel allows capturing more detailed information about the signal's temporal course. After each convolutional layer, the batch normalization is conducted. In deep learning methods, the output of one layer is the input of the next, and so forth. If the parameters of one layer change, the distribution of the input values of the next layer also changes. This shift in inputs distributions, or covariance shift, can be problematic in deep learning methods with many layers (36). Batch normalization mitigates the covariate shift ensuring the normalization of the activations of each layer (36). The batch normalization is followed by the application of the ReLU function. Layers 3, 6, and 9 are subsampling max-pooling layers (MaxPool2D), which also aim to reduce the number of network parameters. As the network expands, the number of channels used is increased to improve network capacity. Sixteen channels are initially set for the first set of convolutional layers, being duplicated after each max-pooling layer from there onwards up to the 9th layer.

From this point onwards, the learning process is slightly different, with special attention to the scalp distribution of the EEG amplitude. The 10th layer, with 32 channels, performs 3-point 1D convolutions over the spatial dimension (1x3 kernel), searching patterns in the distribution across the different electrodes. Afterward, 2D convolutions with 3x3 kernels are applied to find correlations between temporal and spatial information. This layer also has 32 channels, and its output is flattened, becoming a 1D structure with 512 points. This transformation is followed by 3 FC layers, which aim to aggregate information from high-level features extracted in preceding layers and determine which features are more strongly correlated with a particular class. The fully connected layers are implemented through linear functions. The last layer is fully connected to all its inputs (128) and the 2 outputs nodes (one for SZ and other for HC). The first two FC layers are followed by the ReLU function. After the last FC layer, the softmax function is applied to determine the probability distribution of the two outputs.

### 2.3. Traditional Machine Learning

There are few publicly available databases of EEG signals from subjects with a diagnosis of SZ. This hampers the comparison of the distinct classical machine learning or deep learning models proposed in the literature. To assess whether our deep network architecture brings advantages over traditional machine learning methods, we tested different algorithms.

Given the versatility of RFs, these algorithms were applied to perform the SZ classification using features extracted from auditory ERP data. In fact, RF is a robust classification algorithm. This model consists of a large number of small decision trees, known as estimators. RF combines predictions of the estimators to produce a more accurate prediction. This method performs well with heterogeneous and high-dimensionality features, and its ensemble design allows to compensate for the overfitting of standard decision trees (37).

### 2.4. Ensemble Strategy

The ensemble technique consists of combining multiple models, known as base learners, in order to reduce the generalization error and variance (38). Rather than having a single learner as the best predictor, different models are trained separately and then averaged to produce one optimal predictive model (38). This is the strategy behind RF, which is considered a strong classifier. RF uses bagging (Bootstrap Aggregation) to increase the diversity in training of the ensemble (39) and decision trees as base learners (37). A large set of trees are ensembled, and then averaged (40). Since trees are remarkably noisy and weaker classifiers, they strongly benefit from averaging (40).

In an attempt to improve the generalization ability of the proposed deep learning model, we also adopted an ensemble strategy.

## 3. Experimental Setup

In this section, we detail the EEG dataset used, describing the preprocessing and the transformations applied to prepare data to be used as the model's input. Lastly, we describe the setup of our deep learning and traditional machine learning classifiers.

### 3.1. Data

The experimental EEG recordings used in this study were obtained from a publicly available Kaggle dataset (41), hereinafter referred to as *dataset A*. The use of a small pool of training data may result in an increased risk of model overfitting, and, consequently, of poor generalization to new data. Although some methods can be implemented, such as the *k*-fold cross-validation technique, the increase in data quantity can help to minimize overfitting. For this reason, *dataset A* was extended with EEG data collected by our research team, using a similar sensory task, hereinafter referred to as *dataset B*. The entire dataset used in this study encompasses EEG recordings from 63 HC and 65 SZ subjects [*dataset A*: 32 HC and 49 SZ (17 early illness, 32 chronic); *dataset B*: 31 HC and 16 SZ (all first-episode)]. Each subject dataset comprises EEG segments (one segment per trial). Both databases were merged after statistical analyses showed that differences between the two were not significant (see Supplementary Material).

#### 3.1.1. Data Acquisition and Preprocessing

EEG data were recorded using a 64-channel Active Two Biosemi system (Biosemi, Amsterdam, The Netherlands) during the passive listening task, involving the presentation of 100 sounds: 1,000 Hz, 80 dB sound pressure level (SPL), 50 ms duration tones for *dataset A*, and 680 Hz, 70 dB SPL, 50 ms duration tones for *dataset B*. Individual segments (trials - one per tone presented) were created from continuous recordings with a 3,000 ms duration, time-locked to tone onset with 1,500 ms pre-stimulus and 1,500 ms post-stimulus. For the EEG data collection, subjects performed a basic auditory-motor task composed of three different conditions: auditory-motor condition; auditory-only condition; and motor condition, as described in Ford et al. (42) and Pinheiro et al. (43). For the purposes of the current study, only data from the auditory-only condition were considered. *Dataset A* were acquired in a continuous mode at a digitization rate of 1,024 Hz and referenced off-line to averaged earlobe electrodes. *Dataset B* were collected also in a continuous mode but at a sampling rate of 512 Hz and re-referenced to the averaged mastoid electrodes. Both datasets were preprocessed off-line using EEGLab, a MatLab Toolbox (44). Before preprocessing, *dataset A* was downsampled to 512 Hz so that all data had the same sampling frequency. The EEG data were digitally filtered with a 0.1 Hz high-pass filter, and the outlier channels were interpolated. The trials were normalized with a baseline correction by subtracting the mean amplitude of the −100 to 0 ms pre-stimulus interval to the whole segment. Trials were subjected to the FASTER toolbox for artifact rejection and rejection of outlier single trials (45). This preprocessing procedure is explained in more detail in Ford et al. (42).

### 3.2. Deep Learning Algorithm

#### 3.2.1. Temporal Window

Since the focus of this study was on auditory processing, we selected a shorter time window instead of using the signal from the entire segment. The time window considered as input for the proposed model was shortened to 500 ms of duration, corresponding to a total of 256 time points. EEG segments were extracted from −100 to 400 ms time-locked to sound onset, comprising the expected latencies of the auditory N100 and P200 peaks. Figure 3 provides an example of the 2D structure generated for one trial showing the amplitude for the selected ROI (set of electrodes).

#### 3.2.2. Data Normalization

The amplitude range of EEG recordings varies substantially across subjects, or even within a subject. The normalization of the neural network inputs allows not only comparable measures but also the gradient descent to converge faster. Consequently, the EEG data structures created were transformed before the training phase. The min-max normalization was applied in order to rescale the data. The absolute minimum and maximum values were determined for each training segment and for each electrode. The rescaling was then performed, with the segments' amplitudes varying between the average value of the minima and the average value of the maxima determined for each electrode. A Z-score standardization was then applied allowing data of the entire sample to have zero mean and unit variance. This standardization forces data from both groups of subjects to have the same distribution.

#### 3.2.3. Data Partitioning

Each subject's dataset was composed of a variable number of segments after the removal of segments with artifacts (mean of segments/subject: 94,86 ± 2,27). In total, 5,756 trials from 63 HC subjects and 5,853 trials from 65 SZ subjects were used. A stratified 10-fold cross-validation was performed. The dataset splitting was performed by subject rather than by segment. This procedure ensured that segments of a subject contained in a fold did not leak into other folds. The subjects' datasets were shuffled and split into 10-folds, while ensuring that each fold had the same proportion of SZ and HC subjects' data. In each of the ten iterations, the model was trained using 9-folds, while the remaining fold was used for the test. From the subjects contained in the 9 training folds of each iteration, approximately 90% of them were effectively used for training, while the remaining were used as a validation set. Also, in this split we ensured that the selected subjects contained all their EEG segments (see Supplementary Material for more information on dataset splitting). The validation dataset was used to tune hyperparameters and provide an unbiased evaluation of the model. This division was also stratified according to SZ and HC classes, after shuffling the subjects. As in the folds split, the procedure also ensured that segments of a subject did not leak into other folds, so that segments belonging to a training subject were used neither for validation nor for testing. In each iteration, all data segments contained in the training and validation sets were randomized.

#### 3.2.4. Model Training and Hyperparameters

The training process was performed on 300 epochs. The EEG images of all subjects that belonged to the training set were randomly shuffled. The network was trained with ADAM optimizer (*lr* = 1 × 10^−4^) and a mini-batch of size 4. Regularization techniques are implemented to prevent overfitting. The learning algorithm is modified in order to reduce its generalization error, but not the training error (38). For regularization, a spatial dropout of 0.25 at some convolutions was used, as illustrated in Figure 4. To limit the model's capacity, the *L*^2^ regularization was also implemented by adding a parameter norm penalty of 1 × 10^−4^ to the cost function. The Xavier Glorot uniform (46) was used as initializer of the model's parameters, ensuring the zero-mean and keeping the variance of activations the same across every layer. The cross-entropy of the outputs was calculated concerning the true labels, generating a loss. The negative loss likelihood (NLL) function was used to determine the loss (47). As described before, the model output is a probability of an image label being assigned to the SZ or HC groups. For validation purposes, a probability threshold of 50% was considered to classify the segment as SZ or HC. The accuracy of the model was determined by all EEG images included in the analysis using cross-validation. During the optimization by backpropagation, we saved the optimal model evaluated with the validation data set. The typical duration of model training is about 15 h (90 min/fold).

### 3.3. Traditional Machine Learning Algorithms

#### 3.3.1. Feature Extraction

**Figure 5:**
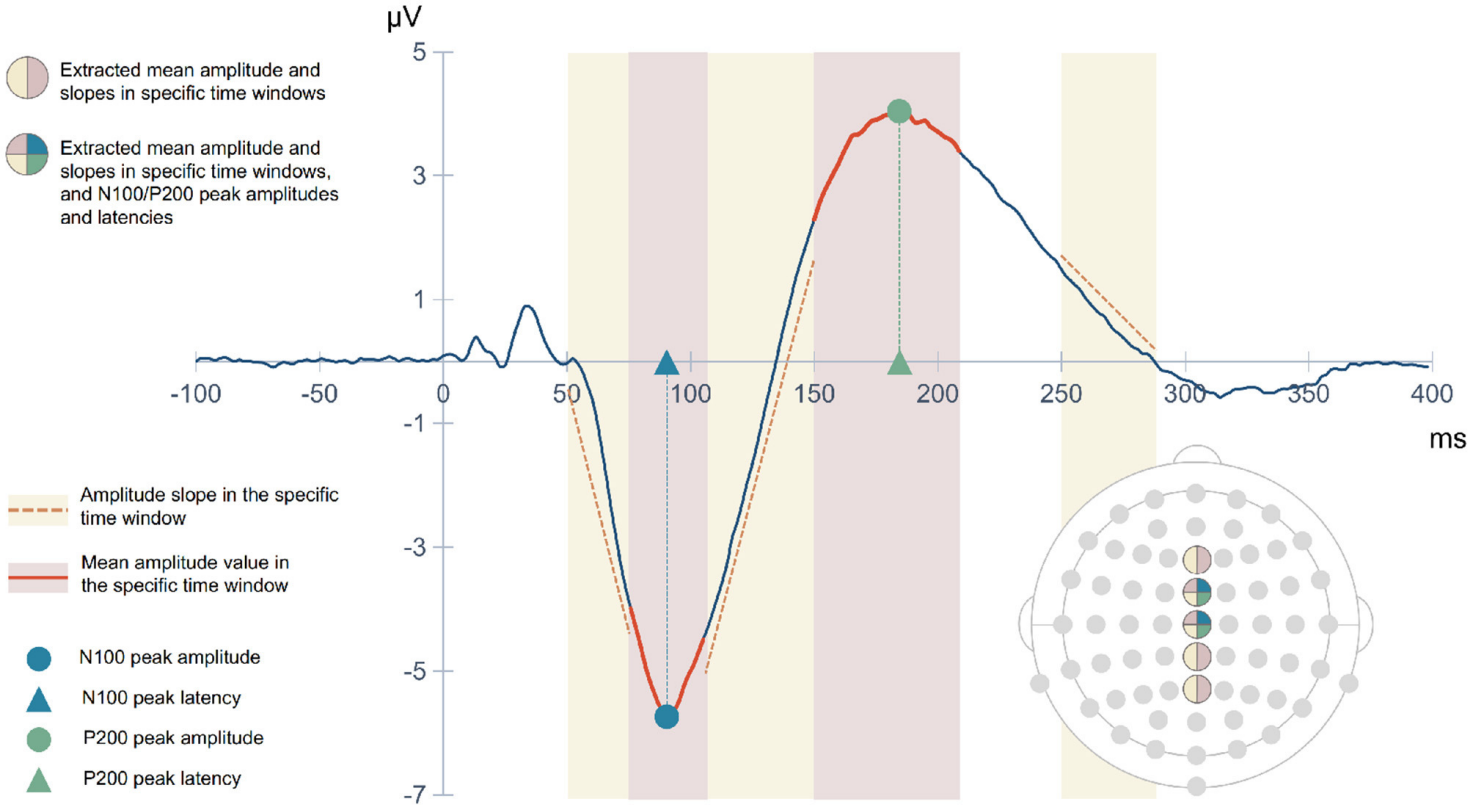
Schematic representation of the features extracted from each subject's ERP waveform.

EEG feature extraction was performed based on ERP waveforms, which were obtained for each subject after averaging across all trials. Figure 5 provides a schematic illustration of the extracted features. The ERP mean amplitude was determined from 75 to 105 ms latency window (post-stimulus onset), corresponding to the window in which the N100 ERP component typically emerges, and from 150 to 210 ms latency window capturing the P200 component. Both time windows are shown with red shading in Figure 5. We also included three slopes as features: the beginning of N100 deflection, the transition from N100 to P200, and the final descending section of the P200 component. Yellow shaded areas in Figure 5 mark the latency intervals considered for calculating these slopes. All these five features were extracted from the signals captured by the electrodes Fz, FCz, Cz, CPz, and Pz. In addition, the amplitudes (circular symbol) and latencies (triangular symbol) of the N100 and P200 peaks (blue and green marks, respectively, in Figure 5) over FCz and Cz electrodes were also extracted. A matrix of features was created, with rows corresponding to the subjects and the columns corresponding to the 33 features extracted for each one.

#### 3.3.2. Data Partitioning and Normalization

A stratified 10-fold cross-validation method was applied to test machine learning models. Subjects (features matrix rows) were shuffled and split into 10-folds, ensuring that each fold had the same proportion of SZ and HC subjects data and that no subject was repeated in any fold. As implemented in the deep learning model, in each of the 10 iterations, the model was trained using 9-folds, while the remaining fold was used for the test. From the subjects contained in the 9 training folds of each iteration, approximately 10% of them were used in the validation set.

#### 3.3.3. Models Training and Hyperparameters

The SZ classification was firstly performed with the RF algorithm using features extracted from the 5 electrodes. Several tests were performed to evaluate the effect of the different extracted features. The hyperparameter tuning was conducted for each one, focused on the number of estimators, maximum number of features, maximum depth of the tree, and split criterion using the validation set. Initially, only the mean amplitudes in the N100 and P200 windows were used, with ten features included per subject. RF configured with 100 estimators, a maximum number of features of 5, and a maximum depth of 2, achieved the best performance. Afterward, the N100 and P200 peak amplitudes and latencies were added to the previous data set, totaling 18 features per subject. The best model was configured with 50 estimators, a maximum number of 10, and a maximum depth of 2. Finally, slopes were also considered (Section 4.4.1 will provide the rationale for including slopes as features). The best model performance using the 33 features per subject was achieved with 100 estimators, using a maximum of 2 features in each split and a maximum depth of 15 in each tree. In both cases, the criterion used to find the optimum split in the validation set was the *Gini* impurity measure.

### 3.4. Ensemble Method

We used the SzNet as base learner and the hard voting algorithm as the ensemble method. We combined 5 fits of the SzNet model trained with different randomly selected seeds in model's weights initialization. This approach is justified by the fact that different initializations lead the neural networks to converge to distinct solutions (48). The hard voting of predictions from separately trained models is one of the simplest ensemble methods and it predicts the class with the largest sum of votes from the models.

### 3.5. Evaluation Metrics

In order to test the models' performance, all input samples of each subject were fed into the model. The probability of each sample belonging to a SZ subject was computed. Unlike validation, a subject-based approach was considered to test the model. Thus, the average of each subject's input samples probabilities was determined and the same probability threshold (50%) was used to assign a label to the subject. This label can be positive (SZ) or negative (HC). To describe the prediction quality, five metrics were derived from the confusion matrix (Table 1).

**Table 1:** Metrics used to assess the model's performance, their formulas, and descriptions.

**Metric**	**Formula**	**Description**
Accuracy	$\frac{TP+TN}{TP+TN+FP+FN}$	Proportion of correctly predicted subjects labels
Recall	$\frac{TP}{TP+FN}$	Proportion of correctly predicted SZ subjects
Specificity	$\frac{TN}{TN+FP}$	Proportion of correctly predicted HC subjects
Precision	$\frac{TP}{TP+FP}$	How consistent predictions are when tests are repeated
AUC-ROC	Area under the curve: *Recall* vs. $\frac{FP}{FP+TN}$	How much the model is capable of distinguishing between classes

*TN, True Negative; TP, True Positive; FN, False Negative; FP, False Positive*.

### 3.6. Implementation Details

Both deep and traditional machine learning models were implemented on a workstation with an Intel® Core™ CPU (i7-4770k, 3.5 GHz) and an NVIDIA® GPU (GeForce® GTX 1070). Deep learning models were implemented using the open-source framework Pytorch, based on Torch library. RF algorithms were also written in python using *Scikit Learn* (49) and *NumPy* (50) packages.

## 4. Results and Discussion

In this section, we start by motivating the choice of kernel for time-domain convolutions. Thereafter, we present and discuss the ablative study, which assesses the relevance of the amount and location of EEG spatial-temporal information used. In this ablative study, we evaluate the number of electrodes used as well as their location over the scalp. After, we compare our proposal with related work, and we present its limitations.

### 4.1. Kernel Size for Temporal Convolution

The kernel size determines the receptive field of a convolution and provides information about the number of input datapoints the network can look at (51). This is a factor to take into account when considering a network's ability to encode the features, and it is associated with the learning parameters.

In this study, the kernel size used for time-domain convolution defines what information is extracted from the EEG signal. The decomposition of EEG signals can reveal oscillatory activity in specific frequency bands (52). Activity in each band has been associated with different functions. The faster rhythms of the EEG signal, corresponding to the gamma band (above 30 Hz), are linked to complex auditory information processing. Desynchronization of these oscillations during auditory processing has been reported in SZ (52, 53). Thus, we search for the best kernel size using cross-validation. After testing various sizes, the 9x1 kernel was the one that reflected the best results.

### 4.2. Ablation Study

#### 4.2.1. Relevance of the Number and Location of Electrodes

**Figure 6:**
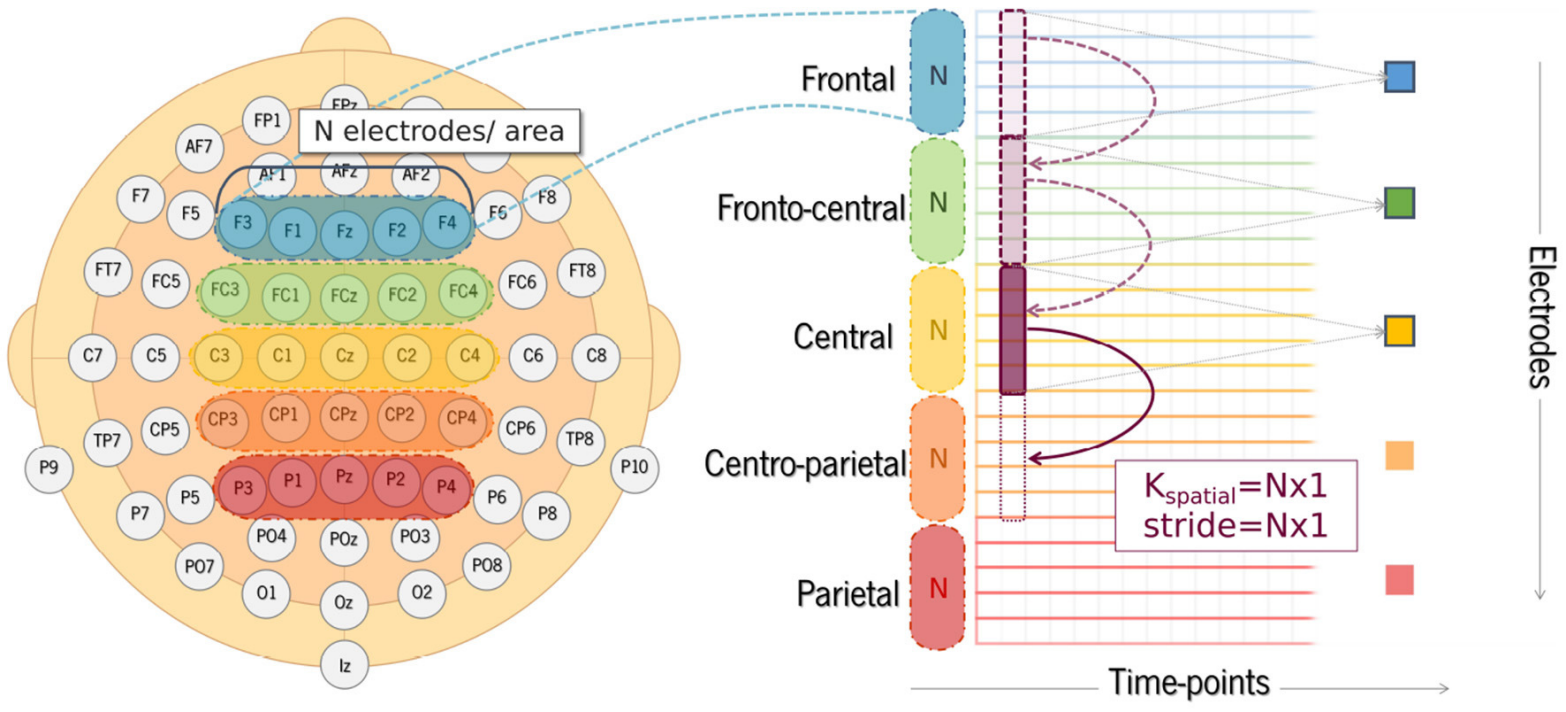
Illustration of the electrode alignment in the 2D structure created for each EEG segment, and schematization of the convolutional process of the extra layer added to the SzNet-15 and SzNet-35 networks. *K*_spatial_ represents the kernel used in the convolutional layer. *N* corresponds to the number of electrodes of each area tested.

Different regions of interest (ROI) were analyzed in order to understand whether and which spatial information is relevant for SZ discrimination. The effects of the number of electrodes and their location were tested. The choice of specific electrodes considered the topographical distribution found both for N100 and P200 ERP components (Figure 2). Since the largest amplitudes of these components are widely spread over the frontal, central, and parietal areas, the first approach used all the 35 electrodes covering these regions. Then, the number of electrodes was reduced, keeping the coverage of the same scalp areas, but varying its location from the left hemisphere to the right. Three combinations of electrodes were considered (5, 15, and 35 electrodes over the frontal, fronto-central, central, centro-parietal, and parietal regions). The three models are hereinafter referred to as SzNet-5, SzNet-15, and SzNet-35 corresponding to the three sets used: 5, 15, and 35-electrodes sets, respectively. To assess the use of 15 and 35 electrodes, the SzNet architecture was slightly changed: an extra convolutional layer (with 32 filters) was incremented after layer 9, followed by a ReLU function (additional details on the architectures of these models are provided in the Supplementary Material). The electrodes in 2D structures were also aligned from the frontal to the parietal region, and from the left to the right hemisphere, as exemplified in Figure 6. *N* corresponds to the number of electrodes from each scalp region tested, which was set to 3 for SzNet-15 and to 7 for SzNet-35. The kernel size used in the extra convolutional layer is dependent on *N*. This layer aimed to gather information from each region (frontal, fronto-central, central, centro-parietal, and parietal). Convolutions with stride equal to *N* in the spatial dimension allowed obtaining one feature per region, as illustrated in Figure 6. The remaining network was unaltered.

**Figure 7:**
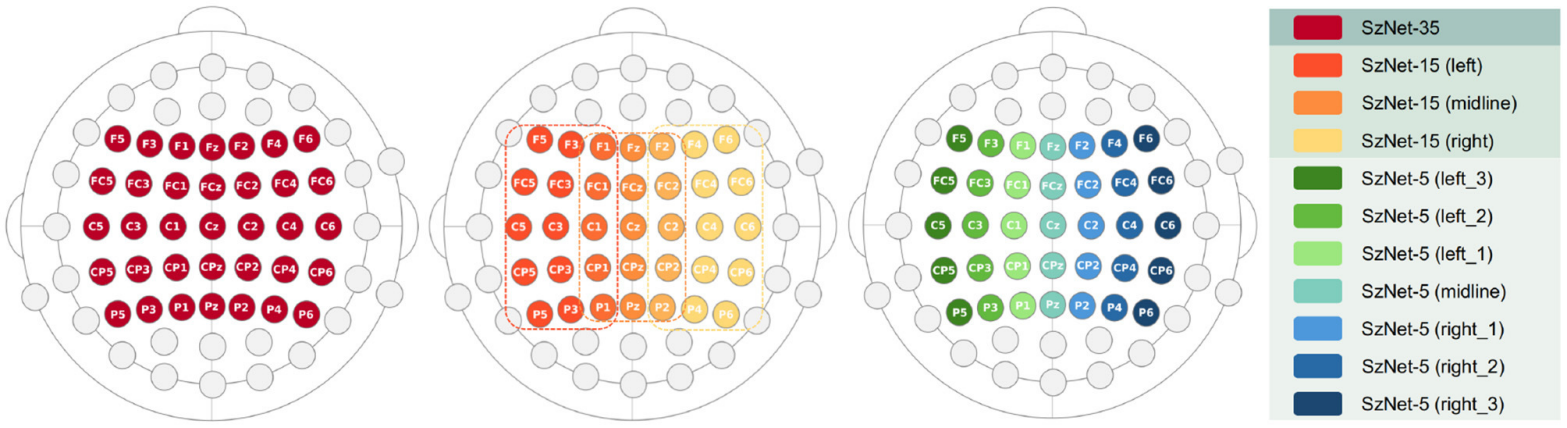
Schematic representation of the different ROIs showing the topographical distribution of electrodes considered to test the three models SzNet-35, SzNet-15, and SzNet-5.

Figure 7 schematically presents the ROI, which varies in number of electrodes and hemispheric location, used to evaluate the performance of the three models developed. The 10-fold cross-validation metrics, computed for each subset of electrodes, are presented in Table 2. The results of SzNet-5 and SzNet-15 using different spatial subsets show differences in accuracy as a function of electrode location in the training and data sets. The training and testing with midline and right ROI data showed improved performances. Both accuracy and AUC-ROC metrics show that SzNet-5 with midline and right-1 data, and SzNet-15-midline achieved the best performances. The lower result achieved by the SzNet-5 left-1 subset can account for the slightly lower performance obtained by SzNet-15-midline.

**Table 2:** Performance of the models tested (SzNet-5, SzNet-15 and SzNet-35) using different spatial ROI evaluated with five metrics: accuracy; AUC-ROC; precision; recall; and specificity.

**Model**	**ROI**	**Accuracy**	**AUC-ROC**	**Precision**	**Recall**	**Specificity**
SzNet-35	-	0.71 ± 0.07	0.71 ± 0.08	0.70 ± 0.7	0.77 ± 0.09	0.65 ± 0.11
SzNet-15	Left	0.67 ± 0.09	0.67 ± 0.09	0.67 ± 0.14	0.79 ± 0.15	0.55 ± 0.26
SzNet-15	Midline	0.76 ± 0.07	0.76 ± 0.07	0.79 ± 0.13	0.77 ± 0.17	0.74 ± 0.19
SzNet-15	Right	0.73 ± 0.10	0.72 ± 0.10	0.69 ± 0.10	**0.88** **±** **0.15**	0.56 ± 0.19
SzNet-5	Left3	0.71 ± 0.10	0.70 ± 0.10	0.70 ± 0.08	0.77 ± 0.19	0.63 ± 0.17
SzNet-5	Left2	0.72 ± 0.05	0.71 ± 0.04	0.75 ± 0.11	0.72 ± 0.20	0.71 ± 0.17
SzNet-5	Left1	0.67 ± 0.08	0.67 ± 0.08	0.71 ± 0.13	0.70 ± 0.20	0.65 ± 0.24
**SzNet-5**	Midline	**0.78** **±** **0.08**	**0.78** **±** **0.08**	**0.82** **±** **0.13**	0.77 ± 0.15	**0.79** **±** **0.17**
SzNet-5	Right1	0.77 ± 0.08	0.77 ± 0.09	0.81 ± 0.21	0.72 ± 0.21	0.77 ± 0.09
SzNet-5	Right2	0.72 ± 0.11	0.72 ± 0.11	0.70 ± 0.10	0.78 ± 0.19	0.65 ± 0.14
SzNet-5	Right3	0.67 ± 0.07	0.67 ± 0.07	0.70 ± 0.14	0.72 ± 0.23	0.61 ± 0.26
SzNet-5	Midline^**^	0.77 ± 0.09	0.77 ± 0.09	0.86 ± 0.16	0.68 ± 0.15	0.85 ± 0.16

*The highest values achieved for each metric are highlighted in a different color. Similarly, the model and ROI that achieved the best results are marked*.

** *randomly aligned ROI*.

Although no asymmetries in auditory processing alterations have been reported in SZ patients (54), these results suggest that changes in amplitude of right hemisphere EEG signals are contributing more to the discrimination of SZ and HC subjects. Nonetheless, the use of midline electrodes seems more beneficial and is corroborated by most of N100 studies in SZ, which often report a medial to midline N100 amplitude decrease (14). The results suggest that the use of small subsets of electrodes can improve SZ classification.

Our results suggest that the model using 5 midline electrodes (SzNet-5-midline) more accurately predicts whether the EEG signals come from a SZ or a HC subject. Although with minor differences, this model achieved the best performance, on average, for every metric. Subtle differences in accuracy values can represent important practical improvements since a variation of only 0.8% is necessary to indicate or detect the increase or decrease of a unit in the number of correctly identified subjects.

#### 4.2.2. Benefiting From Electrode Alignment

We propose the alignment of electrodes in the 2D data structures created to feed the deep learning model. Instead of randomly stacking the EEG signal segments, we hypothesized that the alignment according to the topographical arrangement of the electrodes could represent a more realistic way to extract spatial information from the data structures. To confirm that network performance benefits from this strategy, a comparison between random and organized arrangements of segments was conducted for the proposed model, SzNet-5, using the midline ROI. The 10-fold cross-validation metrics are presented in Table 2, with the randomly aligned ROI marked with asterisks at the bottom of the table. With the organized alignment, the accuracy, AUC-ROC, and recall metrics were improved. The most notorious difference is in the recall value, which is higher when the signal segments are not randomly stacked. The increase in this metric means that the ratio of diagnosed patients incorrectly identified as healthy subjects decreased, which would be a positive result in clinical diagnosis. This may suggest that the spatial dynamics of brain activity over time may be relevant in characterizing auditory processing in SZ.

### 4.3. Relevance of SzNet

SZ classification based on classical machine learning methods applied to EEG data recorded during auditory tasks has been performed using features extracted from ERP components. Besides requiring expertise, there is a great variability in the extracted features. By contrast, the use of deep learning allowed the SzNet model to automatically learn patterns from EEG single-trials. Despite the heterogeneity of the disorder, the model managed to distinguish whether the signal belongs to SZ or HC considering a short time window focused on early auditory processing stages. Although it may simultaneously be considered a limitation, the fact that the SZ sample is not subdivided according to the different disease subgroups or stages (e.g., first-episode or chronic patients), mimics the heterogeneity that will always exist in a real clinical situation. Another advantage of our approach is the reduced number of trials and the task used for EEG data acquisition. The elicitation of some ERP components such as the MMN, whose features are recurrently used for classification purposes, requires a large number of stimuli and, consequently, is time-consuming. On the contrary, the passive listening task used in our study is simple and manageable in a clinical environment, where diagnosis is expected to be rapidly made. Besides binary classification, another aim of the current study was to understand if auditory processing impairments may contribute to the characterization of SZ, in particular considering alterations in ERP components that have been proposed as SZ biomarkers. Only the study conducted by Aristizabal et al. (28) documented the application of deep learning methods to auditory EEG signals. Their aim was to identify subjects at risk of developing SZ using the time course of EEG signals in response to auditory stimuli. With a shallower architecture combining convolutional and recurrent networks and with less learnable parameters, an accuracy of 72.54% was achieved. The problem addressed as well as the database used differ from ours, therefore the studies are not directly comparable. Still, it should be noted that Ahmedt-Aristizabal and collaborators used the same 5 electrodes (Fz, FCz, Cz, CPz, Pz) for which our ablative study showed an improved performance. The SzNet results indicate that changes in the auditory processing are key features in SZ diagnosis. This shows the need to look at sensory changes that are often neglected in clinical assessment, and supports the addition of a new domain to the Research Domain Criteria (RDoC) framework, focused on sensory impairments in psychiatric disorders (55).

### 4.4. Machine Learning vs. Deep Learning

The application of classical machine learning algorithms allowed us to obtain a base-model to compare with the proposed deep learning model.

#### 4.4.1. Features Selection

The selection of relevant features plays an essential role in machine learning classification. The auditory N100 and P200 components shed light on the sensory processing of sounds, and their latency and amplitude are affected by the level of subject's arousal, alertness, and attention (56). Abnormalities in these components have been consistently reported in SZ patients, which include reduced amplitude of the N100 as well as reduced amplitude and shorter peak latencies of the P200 (57). Moreover, both auditory ERP components have been used to examine sensory and information processing impairments in SZ (58). In light of this, our focus turned to these components, in particular their amplitude and latency. Time windows for the extraction of the N100 and P200 mean amplitudes were based on visual inspection of the grand average waveforms and on previous studies (6). Since the N100 and P200 peak amplitudes are topographically distributed over frontocentral and central regions, as seen in Figure 2, the FCz and Cz signals were considered for the analysis of both peak amplitude and latency.

**Figure 8:**
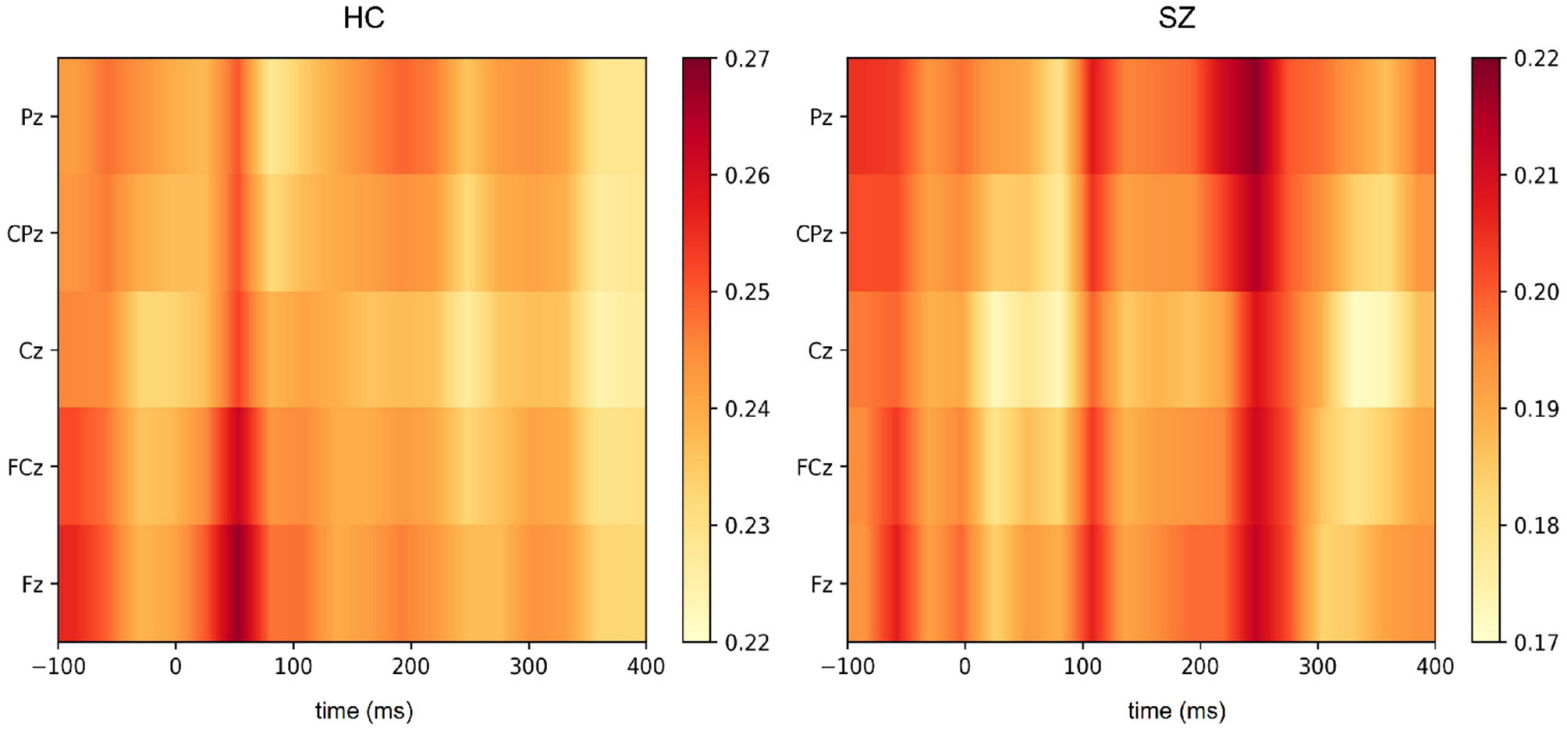
Heatmaps (yellow to red colormap) obtained using Grad-CAM for the segments most likely to belong to HC and SZ subjects.

While auditory processing abnormalities in SZ have been investigated by means of ERP analyses, our deep learning approach extracts features from the signal that may be directly correlated to those components and that are relevant to the discrimination of SZ and healthy subjects. Therefore, the interpretation of the feature extraction in the implemented SzNet model provides more information about their relationship with specific ERP components. The Grad-CAM (Gradient-weighted Class Activation Mapping) algorithm was used to produce a saliency map of the critical regions in the input EEG segments for the classification (59). The importance of the features extracted by the SzNet model (considering the last convolutional layer features) is represented by heatmaps obtained by Grad-CAM implementation in Figure 8. The color scale shows the variation of features relevance, with dark tones representing highly discriminative features in the signal morphology.

For this purpose, we considered the EEG segments correctly identified with a greater degree of certainty (probabilities above 0.80). An averaged heatmap of the selected samples was computed for SZ and HC (Figure 8). An average of the EEG segments was also computed to inform on the features' location and correspondence in the spatio-temporal images.

**Figure 9:**
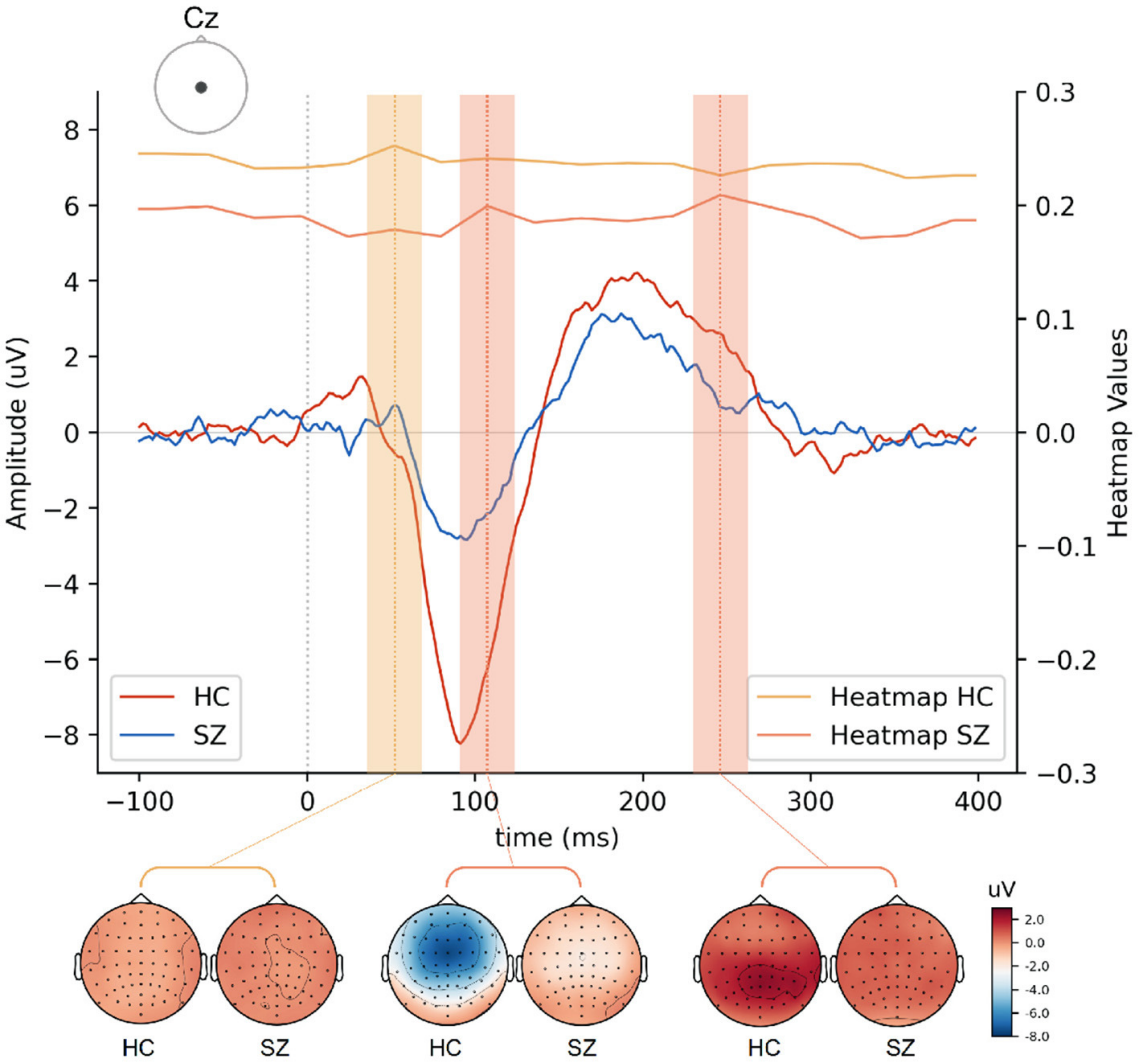
Grad-CAM heatmaps cross-section and averaged EEG signals over Cz electrode drawn for HC and SZ groups. The time intervals associated with the most relevant features are shaded with the color corresponding to the heatmap of each group. Topographical distribution maps of EEG signal amplitudes at those time intervals are also presented for each group.

Figure 9 illustrates HC and SZ heatmaps over Cz and averaged temporal segments (see Supplementary Material for the other electrodes representations). Group differences around 250 ms post-stimulus onset appear to have been the most critical temporal feature for the classification of SZ segments. Interestingly, the transition of more pronounced negative to positive deflections around 110 ms post-stimulus onset, i.e., from the N100 to the P200 deflection, may have been pivotal for SZ identification. This transition is more abrupt in HC as a result of increased N100 and P200 amplitudes. Both salient features seem to have a more significant effect across the five electrodes (Figure 8). The feature extracted from HC segments seem to be more prominent (higher heatmap values) for the classification by inspecting both heatmaps. It should be noted that the most important features to identify HC are located around 50 ms post-stimulus onset. Subtle differences in this time interval may correspond to the transition from a P50-like deflection, whose latency seems to vary between groups, to a N100 deflection. The transitions from P50 to N100, N100 to P200, and the P200 end seem to have relevance for the discrimination of the two groups. In order to interpret these findings, we also extracted the slopes in the time intervals corresponding to those three transitions as input features for machine learning models.

**Figure 10:**
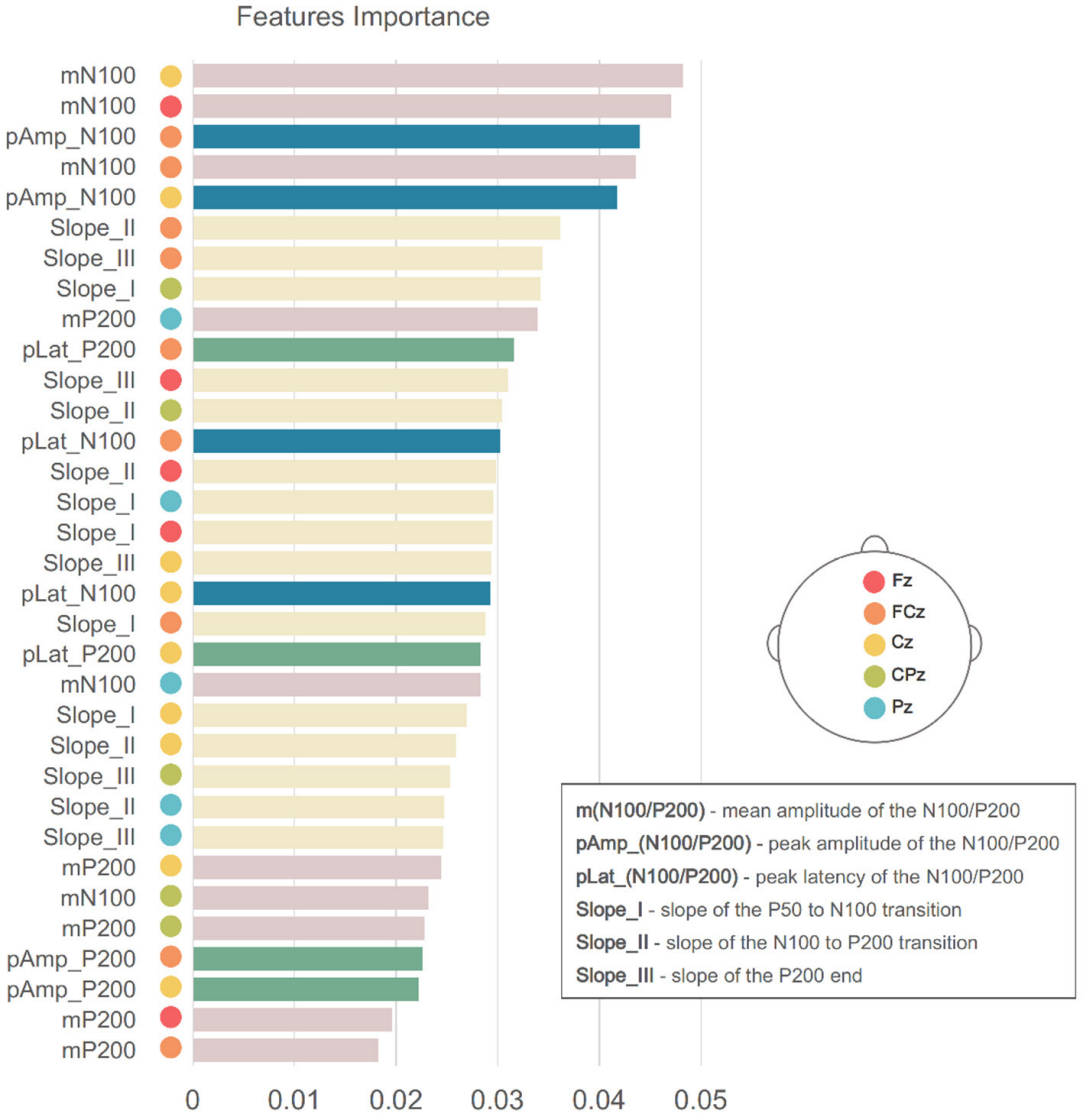
Importance of each feature given by its weight to final performance of RF using 33 features. Bars colors corresponds to the colors used in Figure 5.

RF can provide a measure of the importance of each feature, according to its contribution to the overall classification performance. Benefiting from the increased system interpretability, the features' weights may provide important information on the critical EEG electrodes and features for discriminating SZ. Figure 10 shows the weight of each feature for the RF result considering 33 features. N100 features from frontal, fronto-central, and central scalp regions were the most important features for classification, which is consistent with the literature (14). However, the slopes extracted from the transitions between ERP components appear to have been significant. This result suggests that the temporal dynamics of early auditory processing is altered in SZ. Hence, all these features were considered to obtain the best performance as they seem to have an impact on the differentiation of subjects with SZ.

#### 4.4.2. Models Performance

Table 3 presents the RF models' performance using ERP features for SZ classification. The inclusion of amplitude and latency of the N100 and P200 peaks over FCz and Cz electrodes (RF-18) improved the classification achieved by the RF, which only include the N100 and P200 mean amplitudes over all the 5 midline electrodes (RF-10). The best result was obtained when adding all 33 features (RF-33), which included the slopes in the three transition time intervals between ERP components suggested by the GRAD-CAM implementation. The best model distinguished HC and SZ subjects with an accuracy of 73%. Adding the slopes values as features increased the specificity, which suggests that these features may have a more significant influence on the identification of HC subjects.

**Table 3:** Results of the classification performed by the RF, the proposed deep learning model, and the ensemble learning method.

**Model**	**Features**	**Accuracy**	**Precision**	**Recall**	**Specificity**	**AUC-ROC**
RF	10	0.69 ± 0.10	0.70 ± 0.15	0.70 ± 0.17	0.69 ± 0.14	0.70 ± 0.10
RF	18	0.70 ± 0.11	0.71 ± 0.14	0.70 ± 0.17	0.71 ± 0.15	0.70 ± 0.11
RF	33	0.73 ± 0.13	0.73 ± 0.15	0.70 ± 0.17	0.76 ± 0.13	0.73 ± 0.13
**Proposed**		0.78 ± 0.08	0.82 ± 0.13	0.77 ± 0.15	0.79 ± 0.17	0.78 ± 0.08
**Ensemble**		0.80 ± 0.08	0.82 ± 0.14	0.82 ± 0.14	0.78 ± 0.18	0.80 ± 0.08

The performance of the deep learning method surpasses that of the traditional machine learning models, and demonstrates the potential of the model we propose here for SZ classification. Unlike standard techniques in which features are extracted manually and provided to the model for classification, the deep learning model performs both feature extraction and classification. CNNs are able to detect highly specific features of the training dataset under the constraints of the specific prediction, such as the data labels (38). The end-to-end training forces feature extraction by minimizing the loss for SZ classification. Through this optimization technique, CNNs provide improved models and, thus, more accurate results (38).

### 4.5. Ensemble Learning

The result of the ensemble of SzNet models' predictions are presented in Table 3. An improved performance is observed using 5 models, surpassing the single SzNet result (see Supplementary Material). As expected, the voting ensemble offered a lower variance in predictions made over base learners, which improved the generalization ability.

### 4.6. Limitations and Future Directions

The single-trial EEG recordings contain task-related activity. However, this activity is overlaid with task-unrelated brain processes, which results in a low signal-to-noise ratio (SNR). Variations in SNR across trials and subjects, which may also arise from differences in EEG data acquisition parameters, may affect signal quality. Moreover, fluctuations in the latency and magnitude of the EEG responses to stimuli, combined with the high dimensionality of EEG signals, increase both intra- and intersubject variability. The best performances from raw EEG data may be achieved with larger training data sets, which also prevents overfitting: more subjects and more trials per subject to overcome inter and intrasubject variability, respectively. Data augmentation techniques may increase the diversity of the data available for training the model, and thus prevent overfitting. Notwithstanding, we note that our attempts at data augmentation did not lead to improved SzNet model performance.

As mentioned before, SZ subgroups or stages (e.g., first episode vs. chronic) were not considered in this study. Future studies should address whether SzNet performance is affected by illness stage and symptom severity. However, some requirements should be taken into account: adaptation of the SzNet model for multi-class classification; inclusion of a larger amount of data from each SZ subgroup. Following the adaptation of the model to multiple classes, an important approach might be to include data from subjects at risk of developing SZ or in prodromal stages. This line of development may allow prediction of disease onset and a better understanding of the course of the underlying auditory processing alterations.

SzNet can provide a baseline model for future developments and analyses. A well-characterized public SZ EEG database is highly recommended for the direct comparison and objective evaluation of the performances of different algorithms.

## 5. Conclusion

This paper presents the application of a deep convolutional neural network to the analysis of EEG signals recorded in a passive listening task in healthy and SZ adults. Using only 5 midline electrodes (Fz, FCz, Cz, CPz, and Pz), the proposed model achieved an average accuracy of 78% in the discrimination between SZ and HC subjects. By ensembling predictions of 5 fits of this model, trained with different weights initialization, SZ classification achieved an accuracy of 80%. The deep network allowed the automatic learning of patterns from the time course and spatial distribution of EEG single-trials, capable of detecting alterations in brain indices of auditory processing in SZ, despite the great heterogeneity of the disorder. SzNet provides a base model for future developments in SZ research and, specifically, (differential) diagnosis and prediction.

## Data Availability Statement

A publicly available dataset was used in data analysis. *Dataset B* can be found here: https://www.kaggle.com/broach/button-tone-sz.

## Ethics Statement

The studies involving human participants were reviewed and approved by University of California at San Francisco's Human Research Protection Program (HRPP), USA, and by the Ethics Committees of Faculdade de Psicologia da Universidade de Lisboa, Universidade do Minho, and Hospital de Santa Maria, Portugal. The patients/participants provided their written informed consent to participate in this study.

## Author Contributions

CB, AP, and CS: conception and design of study. BR, JF, and AP: acquisition of data. CB: implementation of machine and deep learning models, analysis and interpretation of data, and drafting the manuscript. CS, AP, BR, and JF: revising the manuscript critically for important intellectual content. All authors contributed to the article and approved the submitted version.

## Funding

This research work was supported by Grant SFRH/BD/111083/2015, funded by Fundação para a Ciência e Tecnologia (FCT) under the Programa Operacional Capital Humano (POCH) co-funded by Portugal 2020 and European Social Fund, by Grant PTDC/MHC-PCN/0101/2014 funded by FCT, and by project UID/EEA/04436/2019 funded by FCT through national funds (PID-DAC). It was also funded by an NIMH grant (R01-S MH058262) and a Department of Veterans Affairs (USA) Senior Research Career Award to JF.

## Conflict of Interest

The authors declare that the research was conducted in the absence of any commercial or financial relationships that could be construed as a potential conflict of interest.

## Publisher's Note

All claims expressed in this article are solely those of the authors and do not necessarily represent those of their affiliated organizations, or those of the publisher, the editors and the reviewers. Any product that may be evaluated in this article, or claim that may be made by its manufacturer, is not guaranteed or endorsed by the publisher.
