# Supplementary material for: From Sound Perception to Automatic Detection of Schizophrenia: An EEG-Based Deep Learning Approach
Source: Front Psychiatry. 2022 Feb 17;12:813460. doi: 10.3389/fpsyt.2021.813460 (PMC8892210; doi:10.3389/fpsyt.2021.813460)
Supplement: Supplementary file 1 [file Data_Sheet_1.PDF]

Supplementary Material

1 MERGING OF DATABASES

Given the similarity of the paradigm used for EEG data acquisition, the feasibility of bringing the two datasets together was studied. We conducted an exploratory mass-univariate analysis on all sensors and time points to assess whether differences in EEG signals' amplitude and topographical distribution of both datasets are significantly affected by variations in stimulus frequency and intensity. To examine group spatiotemporal cluster differences between datasets *A* and *B*, a non-parametric clustering-based permutation method was performed with MNE-Python software (Gramfort et al., 2014). The between-subjects study conducted required an *a priori* averaging step, which consisted of averaging across all trials of each subject to produce evoked responses (Maris and Oostenveld, 2007). For the cluster-based tests we performed 1000 permutations, with a significance level of 0.01 and using the threshold-free cluster enhancement (TFCE) method (e=0.2;h=0; (Smith and Nichols, 2009)). The entire temporal segments (-500 to 1500 ms related to stimulus onset) over the 64 electrodes were analyzed, comparing the data of HC and SZ subjects in dataset *A* with those in dataset *B*. The permutation test conducted on HC data revealed differences between HC-*A* and HC-*B*, although only in F6 electrode at 141 and 143 ms, and in CP6 at 150 ms post-stimulus onset (p¡.01). The cluster-based permutation conducted on SZ data showed that the differences between SZ-*A* and SZ-*B* were smaller than those detected in the HC data, and no difference was statistically significant (p¡.01). The negligible differences between SZ subjects of both datasets suggest that the features of the sounds emitted in the two tasks had no significant effect on the amplitude of these subjects' brain responses. Assuming this, the differences found in the HC data can be explained by high heterogeneity in this group of subjects.

2 DATA PARTIONING

As the remainder is non-zero after dividing the total number of subjects by the 10 folds, the number of subjects per fold is variable. Consequently, the number of subjects in the training and validation sets is also variable. Figure S1 schematically presents the the distribution of subjects across training, validation and test sets in each iteration. One subject belonged to only one fold in each iteration, ensuring that this subject's data did not spill over into the other folds. This data partitioning was employed in both the conventional machine learning and deep learning approaches.

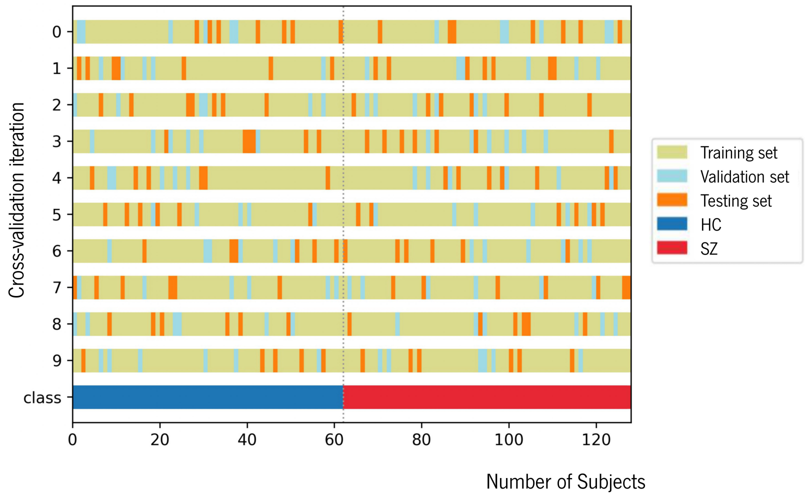

Figure S1. Stratified 10-fold cross-validation: balanced split of SZ and HC subjects data into training, validation and testing sets.

3 MODELS ARCHITECTURES

The architectures of SzNet-5, SzNet-15, and SzNet-35 models are presented in Table S1.

Table S1. Architectures of the SzNet model adapted for the three spatial approaches tested.

Layers	SZNet-5					SZNet-15					SZNet-35				
	type	#filters	kernel	stride	input	type	#filters	kernel	stride	input	type	#filters	kernel	stride	input
1	Conv2D	16	9x1	1x1	256x5x1	Conv2D	16	9x1	1x1	256x15x1	Conv2D	16	9x1	1x1	256x35x1
	Dropout	-	-	-	-	Dropout	-	-	-	-	Dropout	-	-	-	-
2	Conv2D	16	9x1	1x1	248x5x16	Conv2D	16	9x1	1x1	248x15x16	Conv2D	16	9x1	1x1	248x35x16
3	MaxPool2D	-	2x1	2x1	240x5x16	MaxPool2D	-	2x1	2x1	240x15x16	MaxPool2D	-	2x1	2x1	240x35x16
4	Conv2D	32	9x1	1x1	120x5x16	Conv2D	32	9x1	1x1	120x15x16	Conv2D	32	9x1	1x1	120x35x16
	Dropout	-	-	-	-	Dropout	-	-	-	-	Dropout	-	-	-	-
5	Conv2D	32	9x1	1x1	112x5x32	Conv2D	32	9x1	1x1	112x15x32	Conv2D	32	9x1	1x1	112x35x32
6	MaxPool2D	-	2x1	2x1	104x5x32	MaxPool2D	-	2x1	2x1	104x15x32	MaxPool2D	-	2x1	2x1	104x35x32
7	Conv2D	64	9x1	1x1	52x5x32	Conv2D	64	9x1	1x1	52x15x32	Conv2D	64	9x1	1x1	52x35x32
	Dropout	-	-	-	-	Dropout	-	-	-	-	Dropout	-	-	-	-
8	Conv2D	64	9x1	1x1	44x5x64	Conv2D	64	9x1	1x1	44x15x64	Conv2D	64	9x1	1x1	44x35x64
9	MaxPool2D	-	2x1	2x1	36x5x64	MaxPool	-	2x1	2x1	36x15x64	MaxPool2D	-	2x1	2x1	36x35x64
10	Conv2D	32	1x3	1x1	18x5x64	Conv2D	32	1x3	1x3	18x15x64	Conv2D	32	1x7	1x7	18x35x64
11	Conv2D	32	3x3	1x1	18x3x32	Conv2D	32	1x3	1x1	18x5x32	Conv2D	32	1x3	1x1	18x5x32
	Flatten	-	-	-	16x1x32	-	-	-	-	-	-	-	-	-	-
	Dropout	-	-	-	-	-	-	-	-	-	-	-	-	-	-
12	FC - 256	-	-	-	512	Conv2D	32	3x3	1x1	18x3x32	Conv2D	32	3x3	1x1	18x3x32
	-	-	-	-	-	Flatten	-	-	-	16x1x32	Flatten	-	-	-	16x1x32
	Dropout	-	-	-	-	Dropout	-	-	-	-	Dropout	-	-	-	-
13	FC - 128	-	-	-	256	FC - 256	-	-	-	512	FC - 256	-	-	-	512
	Dropout	-	-	-	-	Dropout	-	-	-	-	Dropout	-	-	-	-
14	FC - 2	-	-	-	128	FC - 128	-	-	-	256	FC - 128	-	-	-	256
	-	-	-	-	-	Dropout	-	-	-	-	Dropout	-	-	-	-
15	softmax	-	-	-	2	FC - 2	-	-	-	128	FC - 2	-	-	-	128
16	-	-	-	-	-	softmax	-	-	-	2	softmax	-	-	-	2

Abbreviations: Conv - Convolutional layer; Max-pool - Max Pooling; FC - Fully Connected Layer (followed by the number of units).
Note: Input dimensions - (number of time points; number of electrodes; number of feature maps); the grey shadowed area corresponds to the spatial information extraction.

4 DEEP LEARNING MODEL INTERPRETABILITY

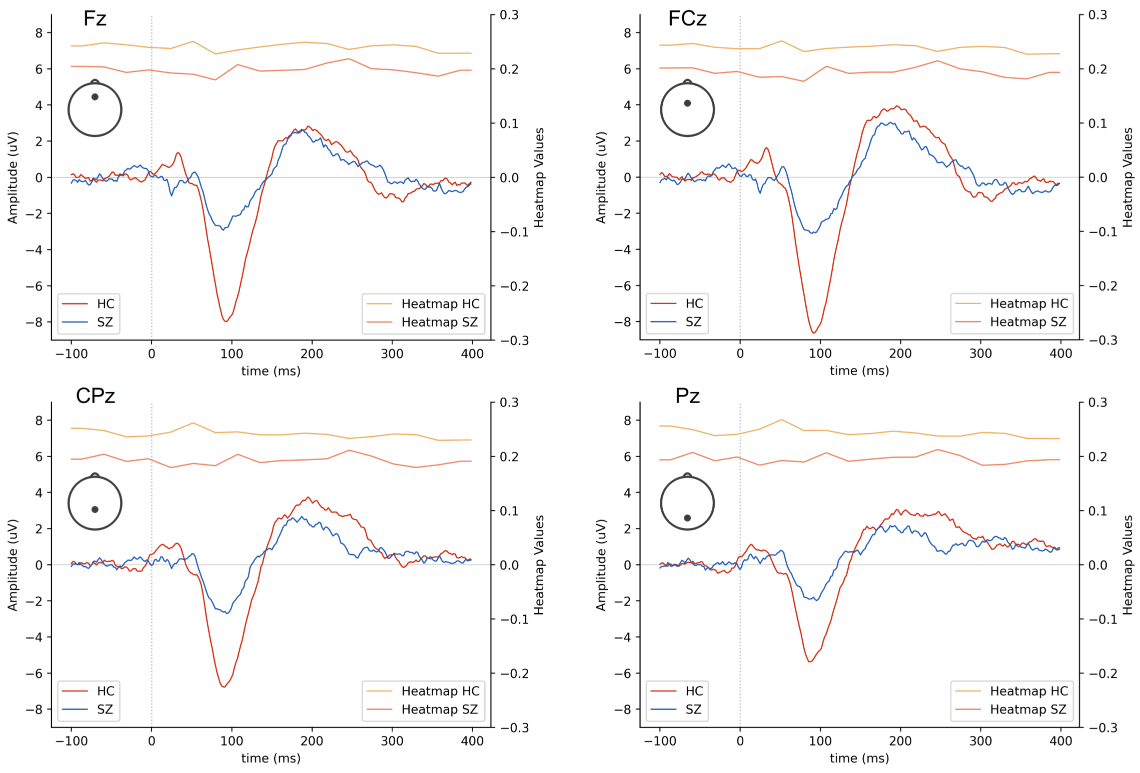

Figure S2. GRAD-CAM heatmaps cross section and averaged EEG signals for Fz, FCz, CPz, and Pz electrodes drawn for HC and SZ groups.

5 ENSEMBLE METHOD

We studied the effect of increasing the number of ensembled models on assessed metrics. By raising the number of models to 7, it was possible to observe an increase in accuracy while the remaining metrics ran differently. The figure presents the results obtained.

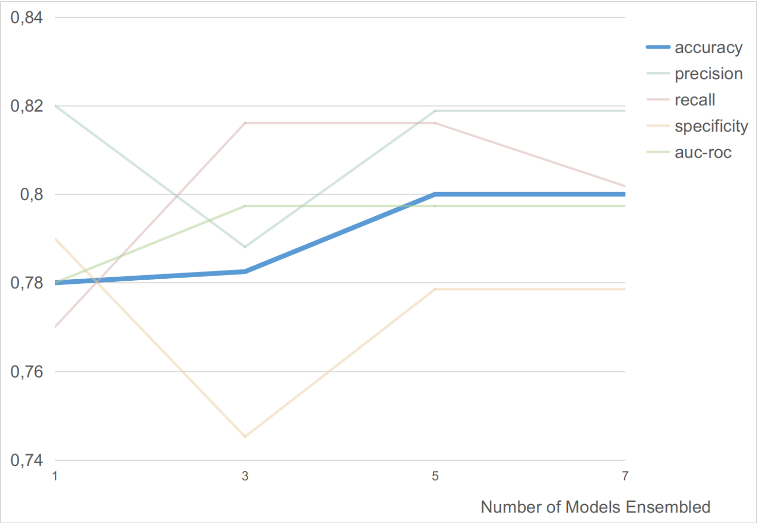

Figure S3. Effects of the growing number of ensembled models on different metrics, with emphasis on accuracy.

REFERENCES

Gramfort, A., Luessi, M., Larson, E., Engemann, D. A., Strohmeier, D., Brodbeck, C., et al. (2014). Mne software for processing meg and eeg data. *Neuroimage* 86, 446–460

Maris, E. and Oostenveld, R. (2007). Nonparametric statistical testing of eeg-and meg-data. *Journal of neuroscience methods* 164, 177–190

Smith, S. M. and Nichols, T. E. (2009). Threshold-free cluster enhancement: addressing problems of smoothing, threshold dependence and localisation in cluster inference. *Neuroimage* 44, 83–98
